# The salivary virome during childhood dental caries

**DOI:** 10.1128/msphere.00198-25

**Published:** 2025-07-28

**Authors:** Jonah Tang, Jonathon L. Baker

**Affiliations:** 1 Department of Biomaterial and Biomedical Sciences, OHSU School of Dentistry 212162 , Portland, Oregon, USA; University of Michigan, Ann Arbor, Michigan, USA

**Keywords:** oral microbiome, bacteriophages, dental caries

## Abstract

While many studies have examined the bacterial taxa associated with dental caries, the most common chronic infectious disease, little is known about the caries-associated virome. In this study, the salivary viromes of 21 children with severe caries (>2 dentin lesions) and 23 children with healthy dentition were examined. A total of 2,485 viral metagenome-assembled genomes (vMAGs) were identified, binned, and quantified from the metagenomic assemblies. These vMAGs were mostly phages and represented 1,865 unique species-level viral operational taxonomic units (vOTUs), of which 478 appear to be novel. The metagenomes were also queried for all 3,858 unique species-level vOTUs of DNA viruses with a human host on NCBI Virus; however, all but *Human betaherpesvirus 7* were at very low abundance in the saliva. The oral viromes of the children with caries exhibited significantly different beta diversity compared to the oral virome of the children with healthy dentition; several vOTUs predicted to infect *Haemophilus* and *Neisseria* were strongly correlated with health, and five vOTUs predicted to infect *Saccharibacteria*, *Prevotella,* and *Veillonella* were correlated with caries. Co-occurrence analysis indicated that the phage typically co-occurred with both their predicted hosts and with bacteria that were themselves associated with the same disease status. Overall, this study provided the sequences of 35 complete or nearly complete novel oral phages and illustrated the potential significance of the oral virome in the context of dental caries, which has been largely overlooked. This work represents an important step toward the identification and study of phage therapy candidates that treat or prevent caries pathogenesis.

**IMPORTANCE:**

Dental caries is the most common chronic infectious disease worldwide and is caused by dysbiosis of the oral microbiome featuring an increased abundance of acid-tolerant, acid-producing, and biofilm-forming bacteria. The oral microbiome also contains viruses; however, very little is known about the caries-associated virome. In this study, the salivary virome of children with severe caries was compared to the salivary virome of children with healthy dentition. The metagenomes contained a total of 1,865 unique species-level viral operational taxonomic units (vOTUs), of which 478 appeared to be novel. The viromes from the children with caries were significantly different than the viromes from the children with healthy teeth, and several health- and disease-associated vOTUs were identified. This study illustrated the potential importance of the oral virome in the context of dental caries and serves as a step towards a better understanding of oral inter-kingdom interactions and identification of potential phage-based caries therapeutics.

## OBSERVATION

The human oral microbiome comprises bacteria, fungi, viruses, archaea, and microeukaryotes and has a major impact on health—with dental caries, periodontal disease, and oral (head and neck) cancers having mainly microbial etiologies (1). Dental caries is the most common chronic infectious disease, characterized by an overabundance of bacterial taxa that are excellent biofilm formers with an ability to generate and tolerate high concentrations of acids, which destroy the tooth enamel (e.g., *Streptococcus mutans*) (2). In addition to its role in oral health, the oral microbiome is increasingly recognized as playing an important role in systemic/overall health (3). The majority of oral microbiome studies have focused on bacteria. In many cases, this is due to the use of 16S rRNA amplicon sequencing, which ignores the non-bacterial constituents. As a result, the study of the virome component of the human microbiome, and its impact, has been very limited despite early landmark studies illustrating that both bacteriophages and human viruses contribute to the health of the oral microbial community and the overall health of the human host (4–6). Our previous study used deep metagenomics to examine the oral microbiome in advanced childhood caries compared to health (7). In that study, taxonomic abundance analysis using MetaPhlAn2 (8) provided some rudimentary insight into the oral virome and even suggested that *Human gammaherpesvirus 4* (Epstein-Barr Virus) was associated with dental caries. However, a specific virus-oriented analysis was not performed, and only bacterial genomes were analyzed from the *de novo* metagenomic assemblies (due to the lack of virus-oriented tools at the time).

**Fig 1:**
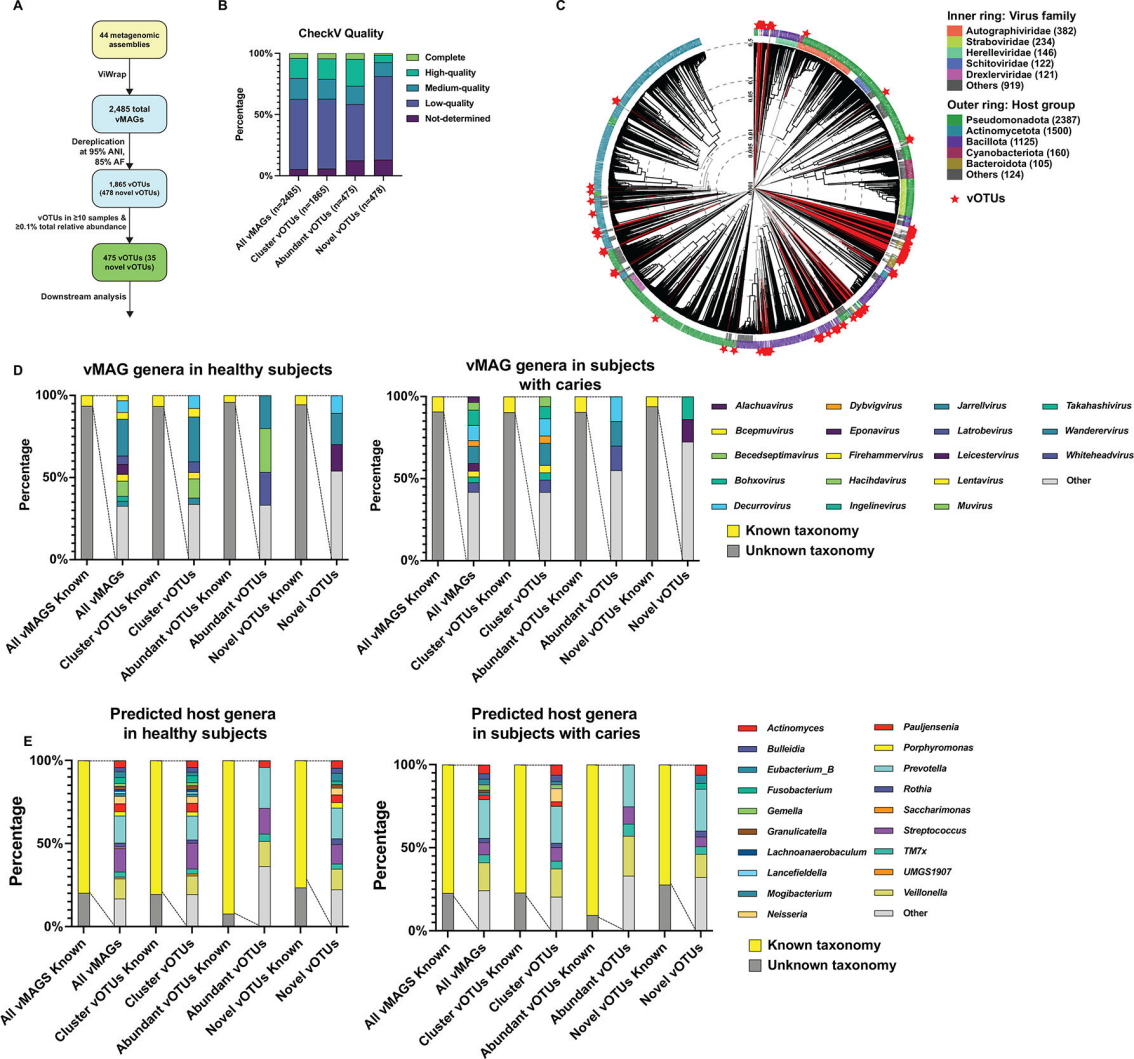
ViWrap analysis of 44 oral metagenomes results in 2,485 vMAGs and 1,865 unique vOTUs, with 478 representing vOTUs not previously described. (**A**) Overview of the approach and viral genomes obtained. Forty-four oral metagenome assemblies were analyzed with the ViWrap pipeline, which identifies and annotates viral sequences, performs binning and quality analysis, and predicts viral and host taxonomy. This resulted in 2,485 vMAGs, which were dereplicated at ≥95% ANI and ≥85% AF using Anvi’o, resulting in 1,865 species-level vOTUs. These vOTUs were compared with all bacteriophage genomes on NCBI Virus, Oral Virus Database, Gut Virome Database, Metagenomic Gut Virus catalog, and IMG/VR4 using skani, which illustrated that at ≥95% ANI and ≥85% AF, 478 of the vOTUs did not have representatives in examined databases and are therefore likely to be novel. Of the vOTUs obtained, 475 were present in ≥10 samples and were at ≥0.1% total relative abundance across all samples, and these were subjected to the downstream analyses. (**B**) vMAG quality. Bar graph illustrating the quality level of all vMAGs, all vOTUs, analyzed vOTUs, and novel vOTUs, as determined by CheckV. (**C**) vOTU phylogeny. Tree generated using VIPTree (13) illustrating the predicted phylogeny of the 300 most abundant contiguous vOTUs from this study, including multi-contig genomes that could be concatenated using ReCCO (14), among viruses in the VIP database, based on protein sequences. The inner ring annotation denotes the predicted taxonomic family of the vOTUs, and the outer ring annotation denotes the predicted host bacteria taxonomic group of the vOTUs. Leaves labeled in red with a star are the 300 most abundant vOTUs derived from this study. (**D**) Predicted vMAG taxonomy based on vConTACT2. Bar graphs illustrating the viral taxonomy of all vMAGs, all vOTUs, analyzed vOTUs, and novel vOTUs. “Other” represents taxonomic units containing ≤2 vMAGs/vOTUs. (**E**) Predicted host taxonomy based on iPHoP. Bar graphs illustrating the taxonomy of bacterial host, predicted by ViWrap, of all vMAGs, all vOTUs, analyzed vOTUs, and novel vOTUs.

Here, 21 assembled oral metagenomes from children with advanced caries and 23 from children with healthy dentition were reanalyzed using the recently developed ViWrap pipeline, optimized for obtaining viral metagenome-assembled genomes (vMAGs) (9) (detailed methods in Text S1; all code associated with this project is available at https://github.com/jonbakerlab/caries-associated-virome). ViWrap identified 2,485 vMAGs across these assemblies (Fig. 1, Table S1). The majority of these vMAGs were viral taxa within the Caudovirales class. Notable exceptions were eight Malgrandavircetes, one Herviviricetes, two Megaviricetes, and one Pokkesviricetes vMAGs. The 2,485 vMAGs were dereplicated to remove redundancy at the cutoffs suggested by the minimum information about an uncultivated virus genome standards (10): ≥95% average nucleotide identity (ANI) and ≥85% alignment fraction (AF), resulting in 1,865 species-level viral genome clusters; the most complete genome from each cluster was selected to represent the species-level cluster as a viral operational taxonomic unit (vOTU) (Fig. 1, Table S2). To determine the novelty of these genomic sequences, the 1,865 vOTUs were compared to all the “Bacteriophages” from NCBI Virus (11) (42,801 genomes, as of April 2024), as well as the Oral Virus Database (48,425 nonredundant virus genomes) (12), Gut Virome Database (33,242 nonredundant virus genomes), Gut Phage Database (142,809 nonredundant virus genomes), Metagenomic Gut Virus catalog (189,680 nonredundant virus genomes), and IMG/VR4 (15,722,824 virus genomes). A total of 478 vOTUs obtained in this study contained zero hits at ≥95% ANI and ≥85% AF across these databases, indicating that these were novel species-level vOTUs (Fig. 1; Table S3). Across vMAGs and vOTUs, about 4% were predicted to be complete and 15% were predicted to be high-quality (Fig. 1B). Auxiliary metabolic genes (AMGs) are viral genes that augment host functions to benefit the viral infection and/or replication processes; many of these genes encode metabolic functions, which can alter the physiology of the host bacteria and consequently the ecology of the microbiota. Across the vMAGs, there were 115 predicted AMGs, with 11 AMGs identified uniquely in caries-associated microbiomes and 50 AMGs identified uniquely in health-associated microbiomes (Table S4).

Because ViWrap was optimized to identify phage and the previous MetaPhlAn2 analysis had also observed human DNA virus genomes in the oral microbiomes (7), we also sought to quantify the abundance of known human viruses in our metagenomes. The 12,482 genomes of DNA viruses with a human host on NCBI Virus (11) (as of March 2024) were also dereplicated to ≥95% ANI and ≥85% AF, resulting in 3,858 vOTUs (Table S5). The metagenomic reads from the 44 samples were mapped to these human vOTUs, which illustrated that most were of very low abundance across our data set (Table S6). Nineteen of the human vOTUs had ≥10,000 total reads mapped across all samples, and these features were added to the *de novo* assembled vOTUs for 1,884 total vOTU features. To determine the relative abundances of these vOTUs across the metagenomic samples, while also normalizing for genome size, the metagenomic reads were mapped to the 1,884 vOTUs using CoverM (https://github.com/wwood/CoverM) (Table S6). Four hundred seventy-six vOTUs with a total relative abundance ≥0.1% across all samples and present in ≥10 samples (Table S6) were used for downstream alpha diversity, beta diversity, and differential abundance analyses. *Human betaherpesvirus 7* was the only human DNA virus from NCBI Virus passing these cutoffs, present in 34 samples and at 0.17% total abundance across all samples. Overall, vOTUs predicted to infect *Prevotella, Streptococcus, Veillonella,* and *Actinomyces/Pauljensenia* were the most abundant in this study, in agreement with phages infecting *Streptococcus* and *Actinomyces* being predominant across previous studies (15) and with high relative abundance of *Prevotella* and *Veillonella* bacteria in this saliva data set (7).

**Fig 2:**
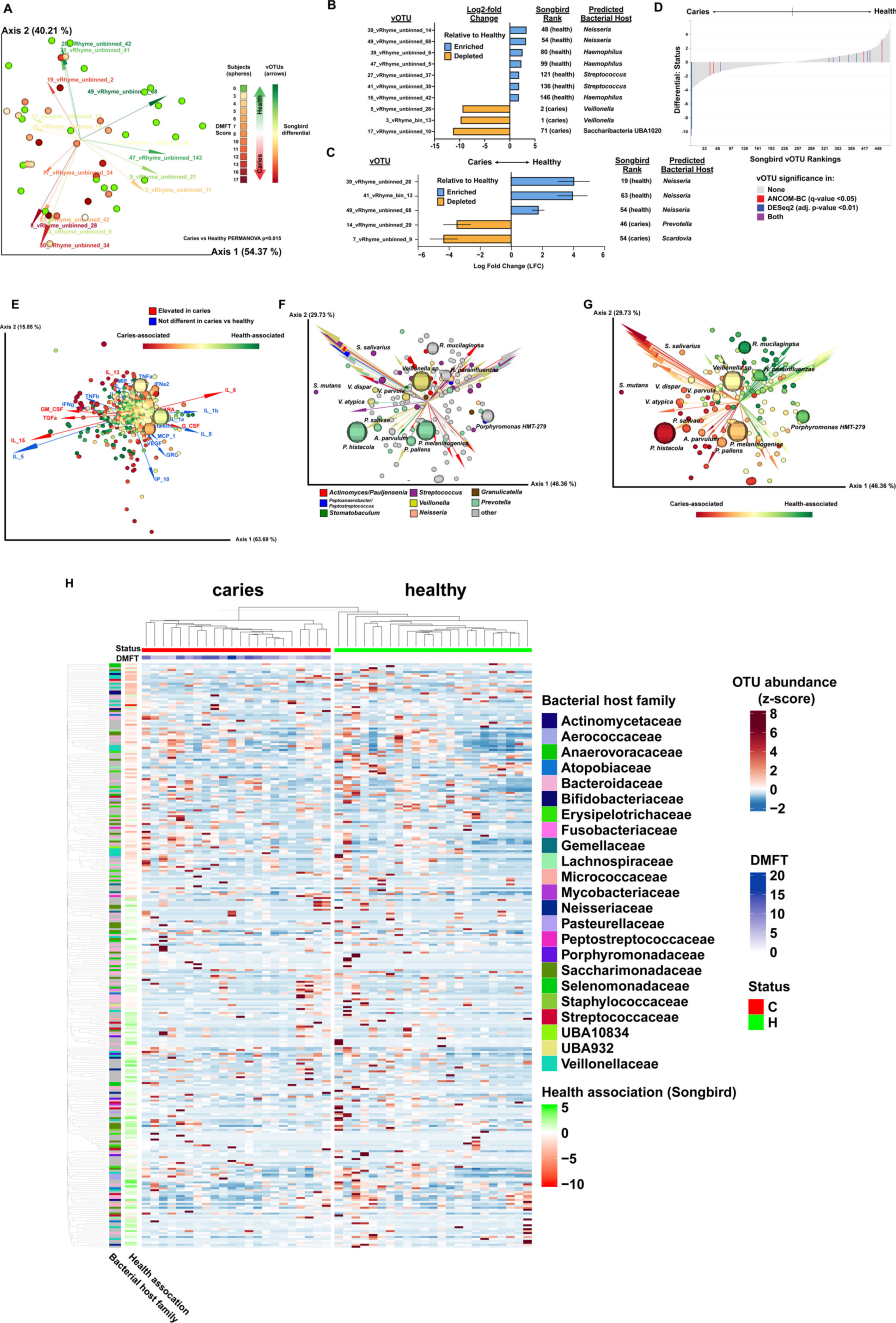
Significant differences in the oral virome and its co-occurrences between healthy children and children with caries. (**A**) Beta diversity. Biplot generated by DEICODE (robust Aitchison PCA) illustrating subjects represented as spheres, which are colored with a gradient indicating the number of decayed, missing, and filled teeth (DMFT) score (i.e., caries severity). Feature loadings (i.e., vOTUs driving the differences in the graph ordination space) are illustrated by the vectors, which are color coded based on their Songbird ranks, indicating association with caries versus health. (**B**) Health- and caries-associated vOTUs identified by DESeq2. Table listing the 10 vOTUs that had relative abundance significantly correlated with either health or caries (adjusted *P*-value < 0.01). “Songbird Rank” indicates the rank of differential from the extreme (i.e., “61 (caries)” indicates the taxon was the 61st in terms of differential associated with caries). (**C**) Health- and caries-associated vOTUs identified by ANCOM-BC. Bar graph illustrating the results of the ANCOM-BC analysis indicating the log fold change of five significant taxa between health and caries (*q*-value < 0.05). (**D**) Bar graph highlighting vOTUs found to be significant by ANCOM-BC (*q*-value < 0.05) and DESeq2 (adjusted *P*-value < 0.01), organized by Songbird feature rankings. Graph generated from Songbird data using Qurro (22). (**E**) Viral-immune marker co-occurrence. Biplot illustrating the co-occurrence of vOTUs with immune markers. vOTUs are represented as spheres, with size indicating total abundance of the taxa across all samples, and color representing Songbird ranks indicating the association of caries versus health. Vectors represent host immune markers, with red vectors indicating host immune markers that were significantly elevated in caries, and blue vectors indicating immune markers that were not significantly different between caries and health, as described in reference 7. (**F and G**) Oral virome-bacteriome co-occurrences. Biplots illustrating the co-occurrence of oral vOTUs with the oral metagenomes (e.g., nearly all bacteria) identified and analyzed in reference 7. Bacterial taxa are represented as spheres, with size indicating the total abundance of the taxa across all samples. vOTUs are represented by the vectors. In panel **F**, colors of the spheres indicate bacterial taxonomy, while colors of the vectors indicate the predicted host taxa of the vOTU (e.g., *Veillonella* bacterial OTUs are yellow spheres, and yellow arrows are vOTUs predicted to infect *Veillonella*). In panel **G**, colors of both the spheres and vectors illustrate Songbird ranking, indicating association with either caries or good dental health (e.g., red spheres are bacterial taxa highly associated with caries, and red arrows are vOTUs highly associated with caries). (**H**) Relative abundance heatmap of vOTUs across samples. Heatmap illustrating the abundance of the 300 most abundant contiguous vOTUs across healthy and caries samples. vOTUs (rows) are clustered based on the phylogeny of protein sequences. Row annotation on the left indicates the predicted host family of the virus, whereas the Songbird differential indicates the association with health versus caries. Interactive QZV files enabling readers to examine the data sets from panels A, E, F, and G in 3D, visualize metadata in different ways, and click on individual data points for more information are available at https://github.com/jonbakerlab/caries-associated-virome.

Beta diversity analysis using the Bray-Curtis, Jaccard, and Aitchison metrics each indicated a significant difference between the viromes of the subjects with caries versus the subjects with healthy teeth (Fig. 2A). No other metadata had a significant impact on beta diversity, and no metadata category had a significant impact on alpha diversity of the viromes. Due to the issues inherent with differential abundance analysis of compositional samples (16), three different approaches were used: Songbird (17) (Table S7), DESeq2 (18) (Table S7), and ANCOM-BC (19). Together, DESeq2 and ANCOM-BC identified nine vOTUs that had increased abundance correlated with healthy dentition, with four predicted to infect *Neisseria,* three predicted to infect *Haemophilus*, and two predicted to infect *Streptococcus* (Fig. 2B). Greater abundance of phages infecting *Haemophilus* in healthy children compared to children with caries has been previously noted (15, 20). The same previous study also identified *Streptococcus* phage M102, which infects *S. mutans*, as significantly elevated in caries (20); however, that specific phage was not identified here. Five vOTUs were identified to be correlated with caries, with two predicted to infect *Veillonella*, and the other three predicted to infect Saccharibacteria, *Prevotella*, and *Scardovia*, respectively (Fig. 2B through D). MMvec (21) was utilized to examine the co-occurrences of the viral features with both the host immunological markers (Fig. 2E, Table S8) and taxonomic features (nearly all bacterial; Fig. 2F and G, Table S8) from the previous study (7). Many of the vOTUs that were most strongly associated with caries had high co-occurrence probabilities with IL-15, TGFα, and GM-CSF, which had elevated concentrations in the saliva of subjects with caries (7) (Fig. 2E). This is in contrast to the bacterial OTUs, where EGF appeared to be a more major factor vis-à-vis caries association (7). Overall, vOTUs tended to co-occur with their predicted bacterial hosts (Fig. 2F). vOTUs also tended to co-occur with bacterial OTUs with a similar Songbird differential ranking with respect to being caries-associated or health-associated (i.e., caries-associated vOTUs and caries-associated bacteria largely occurred together; Fig. 2G).

Altogether, this study illustrated the potential importance of the oral virome in the context of dental caries and the concept that there is still a wealth of unknown viral diversity within the oral microbiome. Limitations of this study include the relatively small and homogenous population size and the fact that many of the vOTUs obtained were incomplete fragments (likely due to processing, DNA extraction, and sequencing methods). Continued improvements in long-read sequencing technology, concurrent with a continued reduction in sequencing costs, will enable larger-scale future metagenomics studies producing more MAGs with greater contiguity. These types of future studies will not only enable an improved understanding of the dynamics of the oral virome and overall microbiome during caries but also hopefully identify promising phages for therapeutic development to treat or prevent this costly disease.

## Data Availability

The raw sequencing reads of the oral metagenomes are available on NCBI with accession numbers PRJNA478018 and SRP151559. The bacterial genomes assembled from those reads were originally described in reference 7 and are available on NCBI under the accession number PRJNA624185. The 35 novel vOTUs rated as either "complete" or "high-quality" by CheckV are currently available at https://github.com/jonbakerlab/caries-associated-virome and at NCBI GenBank with the accession number PRJNA478018. All vMAG sequences, vOTU sequences, scripts, code, and interactive QZV files (enabling readers to examine the data sets from Fig. 2A, E, F, and G in 3D, visualize metadata in different ways, and click on individual data points for more information) are available at https://github.com/jonbakerlab/caries-associated-virome.

## SUPPLEMENTAL MATERIAL

Text S1 msphere.00198-25-s0001.docx Supplemental methods.

Table S1 msphere.00198-25-s0002.xlsx ViWrap vMAGs.

Table S2 msphere.00198-25-s0003.xlsx ViWrap dereplication clusters.

Table S3 msphere.00198-25-s0004.xlsx skani output.

Table S4 msphere.00198-25-s0005.xlsx Predicted AMGs.

Table S5 msphere.00198-25-s0006.xlsx Human DNA virus cluster report.

Table S6 msphere.00198-25-s0007.xlsx OTU tables.

Table S7 msphere.00198-25-s0008.xlsx Differential abundance analysis.

Table S8 msphere.00198-25-s0009.xlsx MMvec conditionals.

ASM does not own the copyrights to Supplemental Material that may be linked to, or accessed through, an article. The authors have granted ASM a non-exclusive, world-wide license to publish the Supplemental Material files. Please contact the corresponding author directly for reuse.
