# Supplementary material for: The salivary virome during childhood dental caries
Source: mSphere. 2025 Jul 28;10(8):e00198-25. doi: 10.1128/msphere.00198-25 (PMC12379589; doi:10.1128/msphere.00198-25)
Supplement: Text S1 — Supplemental methods. [file msphere.00198-25-s0001.docx]

**Supplemental Text S1**

**Supplemental Methods**

**Identification, binning, quality control, and clustering of viral metagenome-assembled genomes (vMAGs).** ViWrap v1.3.0 (1), implementing the Vibrant-VirSorter (vb-vs) identification methods, was used to identify, bin, perform quality control, and predict taxonomy, bacterial host, and auxiliary metabolic genes (AMGs) on viral sequences from 44 assembled oral metagenomes (21 from children with advanced caries and 23 from children with healthy dentition) from NCBI BioProject, PRJNA624185 (2). As described in (2) and (3), raw Illumina reads were subjected to quality filtering and barcode trimming using KneadData v0.5.4 (available at https://bitbucket.org/biobakery/kneaddata) by employing trimmomatic settings of 4-base wide sliding window, with average quality per base >20 and minimum length 90 bp. Reads mapping to the human genome were removed, and metaSPAdes (4) was utilized to *de novo* assemble metagenomes from the quality-filtered Illumina reads. ViWrap analysis resulted in 2,485 total vMAGs (Supplemental Table S1). These vMAGs were dereplicated at ≥95% average nucleotide identity (ANI) and ≥85% alignment frequency (AF) using FastANI (5), implemented in the anvi-dereplicate-genomes function from the active codebase of anvi’o (6). Dereplication resulted in 1,865 species-level genome clusters (Supplemental Table S2). For downstream analysis, a representative vOTU (viral operational taxonomic unit) from each cluster was selected using the metric of highest predicted completeness as determined by ViWrap. To identity any novel vOTUs, the 1,865 vOTUs were compared to six different databases using skani (7): the 42,811 bacteriophage genomes available on NCBI Virus (8) (as of April 2024), all 48,425 genomes in the Oral Virome Database (OVD) (9), all 33,242 genomes in the Gut Virome Database (GVD), all 142,809 genomes in the Gut Phage Database (GPD), all 189,680 genomes in the Metagenomic Gut Virus catalog (MGV), and all 15,722,824 genomes in the IMG/VR4 database (Supplemental Table S3). 478 of the vOTUs from this study did not have a match in a database using ≥95% ANI and ≥85% AF, indicating that these oral vOTUs have likely not been previously described.

**Examining the metagenomes for additional human DNA viruses.** ViWrap was developed to optimally identify phages, rather than human viruses, and previous analysis of these metagenomes using MetaPhlAn2 (10) did identify the presence of several human viruses (and indeed, *Human gammaherpesvirus 4,* [Epstein-Barr virus] was strongly associated with caries) (2). Therefore, all 12,482 available (as of March 2024) human DNA viral genomes were downloaded from the NCBI Virus database (8) and were subsequently dereplicated using anvi’o as described above, yielding 3,858 human DNA virus vOTUs (Supplemental Table S5). For these vOTUs, each cluster representative was selected on the basis of centrality (the anvi’o default). Each metagenomic assembly was mapped to these human DNA vOTUs using BWA MEM 0.7.17-r1188 (11), and the outputs were compiled into a feature table of the human DNA virus vOTUs (Supplemental Table S6). The vast majority of these human DNA vOTUs were at very low abundance, and only the 19 human DNA vOTUs with a total read count of ≥10,000 across all samples were retained for downstream analyses.

**Final OTU table.** To produce an OTU table that was normalized to account for genome length for downstream analysis, the 1,865 ViWrap vOTUs and 19 NCBI vOTUs were concatenated into a single FASTA file, to which the reads from the 45 metagenomes (comprising the aforementioned 44 metagenomic assemblies in addition to one other metagenomic assembly from the original study that did not yield any binned genomes using ViWrap) were mapped using CoverM v0.6.1. The resulting percentage relative abundances for each metagenomic assembly were compiled to construct a relative abundance OTU table (Supplemental Table S6). These relative abundance values were multiplied by a factor of 1,000,000 and rounded to the nearest integer to allow the data to be processed as a frequency OTU table for downstream analyses, with each value substituting as the number of read counts. 476 high abundance features (with a total reads pseudocount of ≥100,000 [i.e., 0.1% total relative abundance across all samples] and present in ≥10 samples; 475 ViWrap vOTUs and 1 NCBI vOTU) were retained for the final OTU table (Supplemental Table S6). Using the methods applied here, *Human gammaherpesvirus 4* was only detected in 2 samples and at 0.0019% total relative abundance, calling into question its association with caries identified in the previous study, which had used clade marker gene-based analysis as opposed to the read-mapping applied here (2). Re-running the latest version of MetaPhlAn that detects viruses (v4.1.0, with the --mpa3 and --add_viruses flags) (12) did not detect significant levels of *Human gammaherpesvirus 4* or *Human betaherpesvirus 7* in sample 5, which had contained the highest abundance of both vOTUs in the original MetaPhlAn2 analysis. This implicates that differences in the MetaPhlAn algorithm and/or databases were responsible for the disparate results between this study and the previous one.

**Downstream analysis.** Alpha (Shannon) diversity metric and beta (Bray-Curtis and Jaccard) diversity metrics and significance were calculated using QIIME 2 (13). Beta diversity using Robust Aitchison PCA was calculated using the DEICODE QIIME 2 plugin (14). Beta diversity visualizations were displayed and manipulated using EMPeror (15). A PERMANOVA calculation with the QIIME 2 beta-group-significance tool indicated that there was a statistically significant difference in beta diversity between the caries and healthy samples using all three metrics (Bray-Curtis, Jaccard, and RPCA). vOTUs associated with caries versus health were identified using the Songbird plugin of QIIME 2 (16) and DeSEQ2 v1.42.1 (17) implemented in R version 4.3.1 (Supplemental Table S7). Multi-omic co-occurrence probabilities of vOTUs and cytokines and co-occurrences of vOTUs and bacteria were calculated using the MMvec plugin for QIIME 2 (18) and visualized using EMPeror (Supplemental Table S8).

**References**

1. Zhou Z, Martin C, Kosmopoulos JC, Anantharaman K. 2023. ViWrap: A modular pipeline to identify, bin, classify, and predict viral-host relationships for viruses from metagenomes. Imeta 2.

2. Jonathon L Baker, James T Morton, Márcia Dinis, Ruth Alvarez, Nini C Tran, Rob Knight, Anna Edlund. 2021. Deep metagenomics examines the oral microbiome during dental caries, revealing novel taxa and co-occurrences with host molecules. Genome Res.

3. Aleti G, Baker JL, Tang X, Alvarez R, Dinis M, Tran NC, Melnik AV, Zhong C, Ernst M, Dorrestein PC, Edlund A. 2019. Identification of the bacterial biosynthetic gene clusters of the oral microbiome illuminates the unexplored social language of bacteria during health and disease. MBio 10.

4. Nurk S, Meleshko D, Korobeynikov A, Pevzner PA. 2017. metaSPAdes: a new versatile metagenomic assembler. Genome Res 27:824–834.

5. Jain C, Rodriguez-R LM, Phillippy AM, Konstantinidis KT, Aluru S. 2018. High throughput ANI analysis of 90K prokaryotic genomes reveals clear species boundaries. Nat Commun 9:5114.

6. Eren AM, Kiefl E, Shaiber A, Veseli I, Miller SE, Schechter MS, Fink I, Pan JN, Yousef M, Fogarty EC, Trigodet F, Watson AR, Esen ÖC, Moore RM, Clayssen Q, Lee MD, Kivenson V, Graham ED, Merrill BD, Karkman A, Blankenberg D, Eppley JM, Sjödin A, Scott JJ, Vázquez-Campos X, McKay LJ, McDaniel EA, Stevens SLR, Anderson RE, Fuessel J, Fernandez-Guerra A, Maignien L, Delmont TO, Willis AD. 2021. Community-led, integrated, reproducible multi-omics with anvi’o. Nat Microbiol 6:3–6.

7. Shaw J, Yu YW. 2023. Fast and robust metagenomic sequence comparison through sparse chaining with skani. Nat Methods 20:1661–1665.

8. NIH. NCBI Virus. NCBI Virus. https://www.ncbi.nlm.nih.gov/labs/virus/vssi/#/. Retrieved 20 March 2024.

9. Li S, Guo R, Zhang Y, Li P, Chen F, Wang X, Li J, Jie Z, Lv Q, Jin H, Wang G, Yan Q. 2022. A catalog of 48,425 nonredundant viruses from oral metagenomes expands the horizon of the human oral virome. iScience 25:104418.

10. Truong DT, Franzosa EA, Tickle TL, Scholz M, Weingart G, Pasolli E, Tett A, Huttenhower C, Segata N. 2015. MetaPhlAn2 for enhanced metagenomic taxonomic profiling. Nat Methods 12:902–903.

11. Vasimuddin Md, Sanchit Misra, Heng Li, Srinivas Aluru. 2019. Efficient Architecture-Aware Acceleration of BWA-MEM for Multicore Systems. IPDPS https://doi.org/10.1109/IPDPS.2019.00041.

12. Blanco-Míguez A, Beghini F, Cumbo F, McIver LJ, Thompson KN, Zolfo M, Manghi P, Dubois L, Huang KD, Thomas AM, Nickols WA, Piccinno G, Piperni E, Punčochář M, Valles-Colomer M, Tett A, Giordano F, Davies R, Wolf J, Berry SE, Spector TD, Franzosa EA, Pasolli E, Asnicar F, Huttenhower C, Segata N. 2023. Extending and improving metagenomic taxonomic profiling with uncharacterized species using MetaPhlAn 4. Nat Biotechnol 41:1633–1644.

13. Bolyen E, Rideout JR, Dillon MR, Bokulich NA, Abnet CC, Al-Ghalith GA, Alexander H, Alm EJ, Arumugam M, Asnicar F, Bai Y, Bisanz JE, Bittinger K, Brejnrod A, Brislawn CJ, Brown CT, Callahan BJ, Caraballo-Rodríguez AM, Chase J, Cope EK, Da Silva R, Diener C, Dorrestein PC, Douglas GM, Durall DM, Duvallet C, Edwardson CF, Ernst M, Estaki M, Fouquier J, Gauglitz JM, Gibbons SM, Gibson DL, Gonzalez A, Gorlick K, Guo J, Hillmann B, Holmes S, Holste H, Huttenhower C, Huttley GA, Janssen S, Jarmusch AK, Jiang L, Kaehler BD, Kang KB, Keefe CR, Keim P, Kelley ST, Knights D, Koester I, Kosciolek T, Kreps J, Langille MGI, Lee J, Ley R, Liu Y-X, Loftfield E, Lozupone C, Maher M, Marotz C, Martin BD, McDonald D, McIver LJ, Melnik AV, Metcalf JL, Morgan SC, Morton JT, Naimey AT, Navas-Molina JA, Nothias LF, Orchanian SB, Pearson T, Peoples SL, Petras D, Preuss ML, Pruesse E, Rasmussen LB, Rivers A, Robeson MS 2nd, Rosenthal P, Segata N, Shaffer M, Shiffer A, Sinha R, Song SJ, Spear JR, Swafford AD, Thompson LR, Torres PJ, Trinh P, Tripathi A, Turnbaugh PJ, Ul-Hasan S, van der Hooft JJJ, Vargas F, Vázquez-Baeza Y, Vogtmann E, von Hippel M, Walters W, Wan Y, Wang M, Warren J, Weber KC, Williamson CHD, Willis AD, Xu ZZ, Zaneveld JR, Zhang Y, Zhu Q, Knight R, Caporaso JG. 2019. Reproducible, interactive, scalable and extensible microbiome data science using QIIME 2. Nat Biotechnol 37:852–857.

14. Martino C, Morton JT, Marotz CA, Thompson LR, Tripathi A, Knight R, Zengler K. 2019. A novel sparse compositional technique reveals microbial perturbations. mSystems 4.

15. Vázquez-Baeza Y, Pirrung M, Gonzalez A, Knight R. 2013. EMPeror: a tool for visualizing high-throughput microbial community data. Gigascience 2:16.

16. Morton JT, Marotz C, Washburne A, Silverman J, Zaramela LS, Edlund A, Zengler K, Knight R. 2019. Establishing microbial composition measurement standards with reference frames. Nat Commun 10:2719.

17. Love MI, Huber W, Anders S. 2014. Moderated estimation of fold change and dispersion for RNA-seq data with DESeq2. Genome Biol 15:550.

18. Morton JT, Aksenov AA, Nothias LF, Foulds JR, Quinn RA, Badri MH, Swenson TL, Van Goethem MW, Northen TR, Vazquez-Baeza Y, Wang M, Bokulich NA, Watters A, Song SJ, Bonneau R, Dorrestein PC, Knight R. 2019. Learning representations of microbe-metabolite interactions. Nat Methods 16:1306–1314.
